# Clinical characteristics and outcome of IgG4-related disease with hypocomplementemia: a prospective cohort study

**DOI:** 10.1186/s13075-021-02481-3

**Published:** 2021-04-07

**Authors:** Linyi Peng, Hui Lu, Jiaxin Zhou, Panpan Zhang, Jieqiong Li, Zheng Liu, Di Wu, Shangzhu Zhang, Yunjiao Yang, Wei Bai, Li Wang, Yunyun Fei, Wen Zhang, Yan Zhao, Xiaofeng Zeng, Fengchun Zhang

**Affiliations:** Department of Rheumatology and Clinical Immunology, Chinese Academy of Medical Sciences & Peking Union Medical College, National Clinical Research Center for Dermatologic and Immunologic Diseases (NCRC-DID), Ministry of Science & Technology; State Key Laboratory of Complex Severe and Rare Diseases, Peking Union Medical College Hospital (PUMCH); Key Laboratory of Rheumatology and Clinical Immunology, Ministry of Education, No.1 Shuai Fu Yuan, Dong Cheng District, Beijing, 100730 China

**Keywords:** IgG4-related disease, Hypocomplementemia, Complement, Immunoglobulin G4, Relapse

## Abstract

**Background:**

Immunoglobulin G4-related disease (IgG4-RD) is a newly recognized systemic, immune-mediated, and fibro-inflammatory disease. Hypocomplementemia was found in part of IgG4-RD patients especially in the setting of active disease.

**Objectives:**

This study aimed to clarify the clinical features, treatment efficacy, and outcome in IgG4-RD patients with hypocomplementemia.

**Methods:**

312 IgG4-RD patients were recruited in our prospective cohort conducted in Peking Union Medical College Hospital. Patients were divided into hypocomplementemia group and normal complement group according to serum C3 and C4 levels measured at baseline before treatment. Low serum C3 levels (< 0.73 g/L) and/or C4 levels (< 0.10 g/L) were defined as hypocomplementemia. Demographic data, clinical characteristics, laboratory parameters, treatment, and outcome of two groups were analyzed and compared.

**Results:**

Hypocomplementemia was identified in 65 (20.8%) cases of untreated IgG4-RD patients at baseline. The average age of hypocomplementemia group was 55.85 ± 10.89 years, with male predominance (72.3%). Compared with normal complement group, patients with hypocomplementemia were likely to have more involved organs, higher IgG4-RD responder index (IgG4-RD RI), and higher laboratory parameters such as counts of eosinophils, inflammatory markers, immunoglobulin G (IgG), IgG1, IgG3, IgG4, and IgE. In addition, lymph nodes, lacrimal gland, submandibular gland, parotid gland, paranasal sinus, bile ducts, and prostate gland were more commonly affected (*p* < 0.05). Serum C3 and C4 showed a significant positively correlation with each other. Both C3 and C4 were negatively correlated with the number of involved organs, IgG, IgG3, IgG4, and IgG4-RD RI, as well as positively correlated with IgA and hypersensitive C reactive protein (hsCRP). 64 (98.5%) patients responded quickly to initial therapy at a 3-month follow-up. Fifteen (23.1%) patients relapsed during follow-up with mean recurrence time of 14.2 ± 13.8 months. Compared with normal complement group, there was no significant difference of relapse rate in two groups (*P* = 0.401).

**Conclusions:**

Clinical characteristics of IgG4-related disease with hypocomplementemia differ from normal complement group. Serum C3 and C4 at baseline before treatment could be biological markers for disease activity. IgG4-RD with hypocomplementemia responded well to treatment and had no significant difference of relapse rate in IgG4-RD with normal complement.

## Introduction

Immunoglobulin G4-related disease (IgG4-RD) is a newly recognized multi-organ, immune-mediated, and fibro-inflammatory disease with pathologically characterized by IgG4-positive lymphoplasmacytic infiltration, storiform-fibrosis, and obliterative phlebitis. IgG4-RD affects nearly every organ, particularly the lacrimal glands, salivary glands, pancreas, bile ducts, lungs, kidneys, retroperitoneum, artery, thyroid gland, meninges, and orbits. Approximately, a quarter to a third of patients with active IgG4-RD have hypocomplementemia defined by the low level of complement component C3 or C4 [1, 2].

Complement is one of the first lines of defense against infections by promoting inflammation and orchestrating opsonization of pathological material, and other critical roles including disposal of immune complexes and apoptotic cellular debris [3]. In addition, it serves as a functional bridge between the innate and adaptive immune systems by enhancing antibody responses and regulating B and T cells activation, playing important roles in the development of numerous inflammatory diseases [3–5]. There are three complement pathways: classical, alternative, and Mannan-binding lectin (MBL) pathways. The cleavage of C3 and C5 leads to the production of the membrane attack complex [5].

Excessive and uncontrolled activation of the complement has been implicated in a series of autoimmune diseases with different pathway and mechanism such as systemic lupus erythematosus (SLE), antiphospholipid syndrome (APS), and anti-neutrophil cytoplasmic antibody-associated vasculitis (AAV). In SLE, the predominant role of the classical pathway in initiation of complement activation, while alternative pathway amplification loop caused complement-mediated damage [4, 6, 7]. In AAV, the alternative pathway and C5a in particular acted as a bridge that links the inflammation and coagulation process [8]. As so far, the role of complement in the pathogenesis of IgG4-RD and which was the activation pathway had not been clarified.

In this study, we focus on clinical features, serum markers, treatment response, and outcome in IgG4-RD with hypocomplementemia; meanwhile, we are trying to investigate whether complement C3 and C4 levels at disease onset could be a biological marker for disease activity and prognosis.

## Methods

### Patient enrollment

In our prospective cohort of IgG4-RD carried out in the Peking Union Medical College Hospital (registered as ClinicalTrials.gov ID: NCT01670695), 312 newly diagnosed patients were enrolled from January 2014 to January 2019, who fulfilled the 2011 comprehensive diagnostic criteria [9, 10], had complement tested at baseline, and had been followed up for more than 6 months. Patients with low serum C3 levels (< 0.73 g/L) and/or C4 levels (< 0.10 g/L) before treatment were defined as hypocomplementemia group. The diagnosis of IgG4-RD was based on the following criteria: (1) a clinical examination showing characteristic diffuse/localized swelling or masses in single or multiple organs, (2) an elevated serum IgG4 concentration (> 135 mg/dL), and (3) a histopathologic examination showing (a) marked lymphocytic and plasma cell infiltration and fibrosis or (b) infiltration of IgG4+ plasma cells (a ratio of IgG4+/IgG+ cells > 40% and > 10 IgG4+ plasma cells per high power field). Patients with other autoimmune diseases, active infection, or malignant disease diagnosed within 5 years were excluded. The study was conducted in compliance with the Declaration of Helsinki and was approved by the Ethics Committee of Peking Union Medical College Hospital (No. S-442). All patients signed written informed consent.

### Clinical data and laboratory parameters

Patients’ data including age, gender, disease duration, history of allergy, treatment strategy, symptom onset, organs affected, and follow-up time were collected. Allergy history was collected using the criteria from the European Academy of Allergy and Clinical Immunology. IgG4-RD responder index (RI) (2018 version) at baseline and each follow-up was evaluated [11] Laboratory parameters included routine blood analysis, liver and kidney function, erythrocyte sedimentation rate (ESR), hypersensitive C-reactive protein (hsCRP); serum complement C3 and C4, serum IgG, A, and M, IgG subclass, total IgE; rheumatoid factor; and auto-antibodies tests. Affected organs and evaluation of treatment efficacy were determined by clinical symptoms, physical examinations, histopathological findings, and imaging, including ultrasonography, computed tomography (CT), magnetic resonance imaging (MRI), or positron emission tomography/computed tomography (PET/CT).

### Assessment of treatment outcomes

Disease response was defined as the decline of the IgG4-RD RI ≥2 points compared with baseline [12]. Clinical relapse was defined as a recurrence of symptoms and signs and/or worsening of imaging studies, with or without re-elevation of the serum IgG4 level [13]. The time of relapse was defined as the date of new onset or recurrence/exacerbation of disease based on symptoms, physical examination, laboratory, or radiology findings after improvement [14].

To compare therapeutic outcomes of hypocomplementemia group and normal complement group, patients who were initially treated with initial GCs alone or GCs plus IMs, with initial GC doses of 0.5–1.0 mg/kg/day (30–60 mg/day) of prednisone equivalent, and followed-up more than 24 months were included.

### Statistical analysis

Statistical analyses were performed using the IBM SPSS Statistics version 24.0 software (IBM, Armonk, NY, USA), the Prism software version 6.1 (GraphPad Software, La Jolla, CA, USA). Data were reported as means ± standard deviation or median and interquartile range (IQR). Normally distributed data between two groups were analyzed using independent-samples *t* tests. Non-normally distributed data were analyzed with Mann–Whitney *U* test. Categorical data were analyzed using the chi-square test. The correlation between serum complement level and laboratory parameters was analyzed with Pearson correlation coefficient in hypocomplementemia group at baseline. Kaplan-Meier survival curves and log-rank tests were used to compare relapse-free survival. Univariate and multivariate Cox regression analysis was performed to estimate the hazard ratio (HR) of relapse for each potential risk factor. *P* values < 0.05 were considered to represent significant differences between two groups.

## Results

### Demographic characteristics of IgG4-RD with hypocomplementemia

In this study, we prospectively enrolled 312 newly diagnosed IgG4-RD patients without treatment, 65 (20.8%) patients had hypocomplementemia (hypocomplementemia group), 244 (78.2%) patients had normal complement (normal complement group), and 3 (1.0%) patients had elevated complement. Of the hypocomplementemia group, 45 (69.2%) cases had both complement C3 and C4 reduction, 14 (21.5%) cases with only C3 reduction, and 6 (9.2%) cases with only C4 reduction. As the number of cases with elevated complement was very small, we mainly compared and discussed hypocomplementemia group and normal complement group. Demographic features of such two groups were shown in Table 1. The age at diagnose in hypocomplementemia patients was 55.85 ± 10.89 years, higher than normal complement group. The median duration of disease prior to initial evaluation was 12 (4, 36) months. There was no significant difference of incidence of allergic history between two groups. Compared with normal complement group, patients with hypocomplementemia showed more number of involved organs (4.88 ± 1.79 vs 2.89 ± 1.36, *P* < 0.001) and higher IgG4-RD RI (15.74 ± 5.78 vs 9.64 ± 4.33, *P* < 0.001) significantly at baseline.

**Table 1:** Comparison of demographic characteristics of IgG4-RD with and without hypocomplementemia at baseline

Characteristics at baseline	Hypocomplementemia Group(*n* = 65)	Normal Complement Group(*n* = 244)	*P-* value
Demographic features			
Gender (male), *n* (%)	**47 (72.3%)**	**144 (59.0%)**	**0.05**
Age of diagnosis, mean ± SD	**55.85 ± 10.89**	**53.05 ± 13.00**	**0.113**
Duration of disease (medium months, IQR)	**12 (4, 36)**	**12 (6, 48)**	**0.131**
Allergy history (*n*, %)	**40 (61.5%)**	**125 (51.2%)**	**0.139**
Number of organs involved (mean ± SD)	**4.88 ± 1.79**	**2.89 ± 1.36**	< 0.001***
IgG4-RD RI, mean ± SD	**15.74 ± 5.78**	**9.64 ± 4.33**	< 0.001***
Organ involvement (*n*%)			
Lymph node	**43 (66.2%)**	**88 (36.1%)**	< 0.001***
Lacrimal gland	**43 (66.2%)**	**111 (45.5%)**	0.003**
Submandibular gland	**41 (63.1%)**	**101 (41.4%)**	0.001**
Pancreas	**33 (50.8%)**	**66 (27.1%)**	< 0.001***
Lung	**33 (50.8%)**	**44 (18.0%)**	< 0.001***
Paranasal sinus	**27 (41.5%)**	**68 (27.9%)**	0.029*
Parotid gland	**22 (33.8%)**	**28 (11.5%)**	< 0.001***
Bile duct	**20 (30.8%)**	**35 (14.3%)**	0.002**
Kidney	**12 (18.5%)**	**26 (10.7%)**	**0.082**
Prostate gland	**10 (15.4%)**	**10 (4.1%)**	0.0218*
Retroperitoneum	**8 (12.3%)**	**46 (18.9%)**	**0.229**
Aorta/artery	**5 (7.7%)**	**28 (11.5%)**	**0.442**
Pituitary	**3 (4.6%)**	**7 (2.9%)**	**0.443**
Gastrointestinal tract	**0 (0.0%)**	**4 (1.6%)**	
Mediastinum	**3 (4.6%)**	**6 (2.5%)**	**0.349**
Thyroid (Riedel’s)	**3 (4.6%)**	**10 (4.1%)**	**0.835**
Serological features			
C3(normal 0.73–1.46 g/L)	**0.54 ± 0.17**	**0.99 ± 0.33**	< 0.001***
C4(normal 0.10–0.40 g/L)	**0.061 ± 0.047**	**0.19 (0.14,0.25)**	< 0.001***
WBC(10^9^/L)	**6.67 ± 1.84**	**6.54 (5.58, 7.75)**	**0.894**
HGB(g/L)	**132.35 ± 18.38**	**134.37 ± 22.07**	**0.508**
PLT(10^9^/L)	**215.80 ± 59.00**	**240.30 ± 75.80**	0.017*
EOS(10^9^/L)	**0.39 (0.14,0.69)**	**0.19 (0.10,0.33)**	0.001**
ESR (mm/H)	**46.34 ± 32.40**	**16 (7, 37)**	< 0.001***
hsCRP (mg/L) (normal< 8mg/L)	**2.5 1(0.82, 8.64)**	**1.86 (0.64, 5.68)**	**0.117**
IgG (normal 7.0–17.0 g/L)	**30.92 ± 15.31**	**18.05 ± 8.79**	< 0.001***
IgA (normal 0.7–4.0 g/L)	**1.59 ± 0.78**	**2.36 ± 1.32**	< 0.001***
IgM (normal 0.4–2.3 g/L)	**0.87 ± 0.63**	**0.81 (0.54, 1.23)**	**0.132**
IgE (KU/L)(normal<60KU/L)	**471.0 (246.75, 880.00)**	**222.00 (63.70, 609.00)**	< 0.001***
IgG1(normal 490–1140 mg/dL)	**1295.11 ± 539.48**	**907.73 ± 439.79**	< 0.001***
IgG2(normal 150–640 mg/dL)	**666.83 ± 542.76**	**612.56 ± 256.77**	**0.259**
IgG3(normal 20–110 mg/dL)	**100.56 ± 80.81**	**50.54 ± 41.23**	< 0.001***
IgG4(normal 80–135 mg/dL)	**2614.13 ± 1915.39**	**547.50 (274.23,1215.50)**	< 0.001***
IgG4/IgG	**0.80 ± 0.44**	**0.44 ± 0.34**	< 0.001***
RF positive (*n*, %)	**20 (*n*** **= 57, 35.1%)**	**44 (*n*** **= 166, 26.5%)**	**0.217**

*IG4-RD RI* IgG4-RD responder index, *WBC* white blood cell count, *HGB* hemoglobin, *PLT* platelet count, *EOS* eosinophil count, *ESR* estimated sedimentation rate, *hsCRP* hypersensitive C-reactive protein, *Ig* immunoglobulin, *RF-positive* the level of rheumatoid factor ≥ 20 IU/ml

*P < 0.05, **P < 0.01, ***P < 0.001

### Comparison of involved organs in hypocomplementemia group and normal complement group

Our data demonstrate the discrepancies in the clinical spectrums between two groups. Compared with normal complement group, patients with hypocomplementemia had significantly higher incidence of lymph node (66.2% vs 36.1%, *P* < 0.001), lacrimal gland (66.2% vs 45.5%, *P* = 0.003), submandibular gland (63.1% vs 41.4%, *P* = 0.001), pancreas (50.8% vs 27.1%, *P* < 0.001), lung (50.8% vs 18.0%, *P* < 0.001), paranasal sinus (41.5% vs 27.9% *P* = 0.029), parotid gland (33.8% vs 11.5%,*P* < 0.001), bile duct (30.8% vs 14.3%, *P* = 0.002), and prostate gland (15.4% vs 4.1%, *P* = 0.021) (Table 1). There was no significant difference in kidney involvement between the two groups.

### Comparison of laboratory parameters in hypocomplementemia group and normal complement group

The average level of serum C3 in hypocomplementemia was 0.54 ± 0.17 g/L (normal 0.73–1.46 g/L) and C4 was 0.061 ± 0.047 g/L (normal 0.10–0.40 g/L)). We further compared the laboratory tests between two groups (Table 1) and found that patients with hypocomplementemia had significantly higher baseline levels of peripheral eosinophils count (median 0.42 × 10^9^/L vs 0.17 × 10^9^/L, *P* = 0.006), ESR (46.34 ± 32.40 mm/h vs median 16 mm/h, *P* < 0.001), IgG (30.92 ± 15.31 g/L vs 18.05 ± 8.79 g/L, *P* < 0.001), total-IgE (median 471.0 KU/L vs 222.0 KU/L, *P* < 0.001), IgG1 (1295.11 ± 539.48 mg/dL vs 907.73 ± 439.79 mg/dL, *P* < 0.001), IgG3 (100.56 ± 80.81 mg/dL vs 50.54 ± 41.23 mg/dL, *P* < 0.001), IgG4 (2614.13 ± 1915.39 mg/dL vs median 547.50 mg/dL, *P* < 0.001), ratio of IgG4/IgG(0.80 ± 0.44 vs 0.44 ± 0.34, *P* < 0.001), whereas significantly lower count of platelet (215.80 ± 59.00 × 10^9^/L vs 240.30 ± 75.80 × 10^9^/L, *P* = 0.017) and IgA (1.59 ± 0.78 g/L vs 2.36 ± 1.32 g/L, *P* < 0.001).

### Correlations between serum C3, C4, and clinical characteristics at baseline

**Fig. 1:**
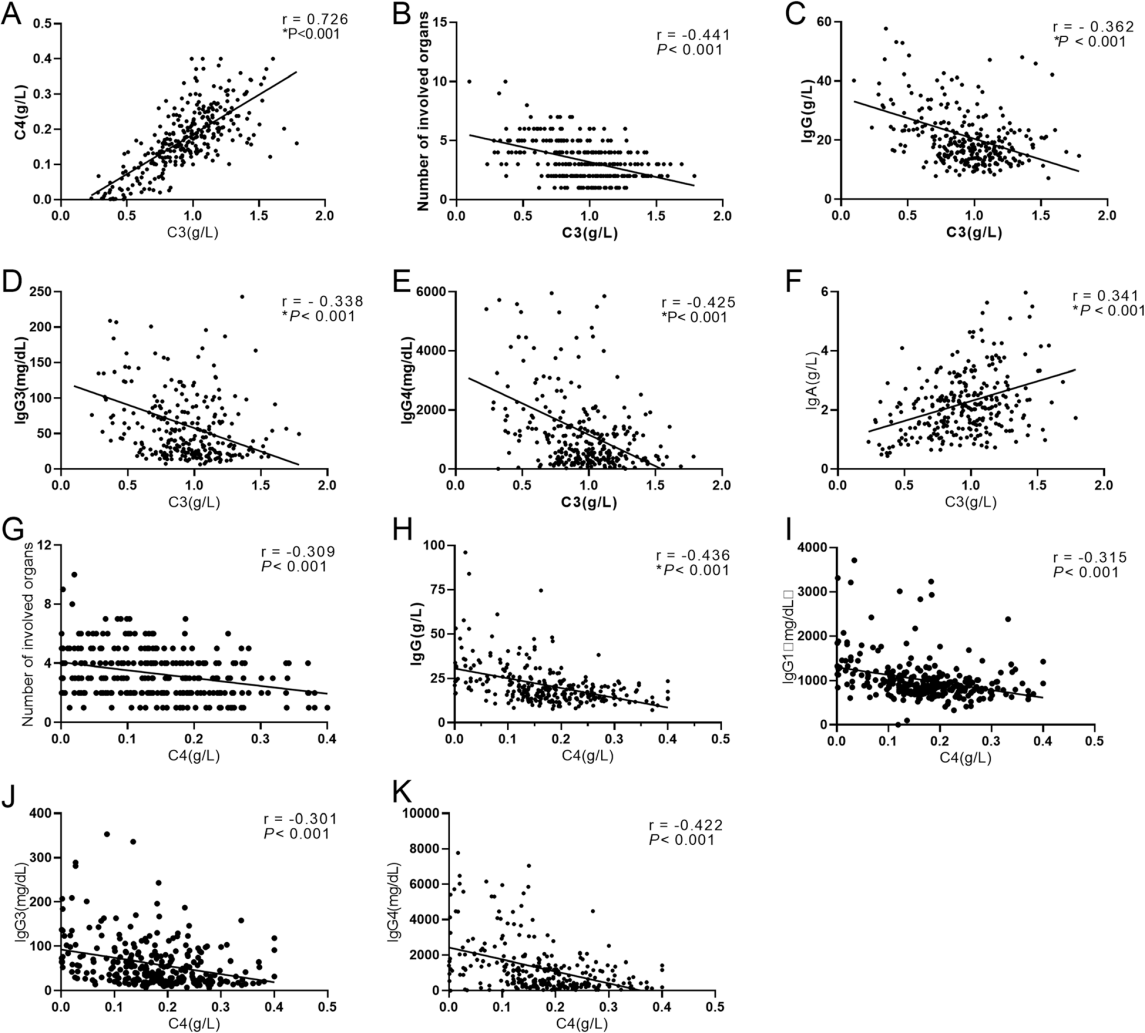
Correlations between baseline serum complement levels and clinical characteristics in IgG4-RD patients. **a–f** Correlations between baseline serum C3 level and clinical characteristics. **g–k** Correlations between serum C4 level and clinical characteristics

We performed Pearson correlation coefficient analysis to investigate the association between serum complement level and age of onset, duration of disease, number of involved organs, IgG4-RD RI, and laboratory parameters including C3/C4, count of eosinophil, ESR, hsCRP, IgG, IgA, IgM, IgE, IgG1, IgG2, IgG3, and IgG4, in total patients at baseline. As shown in Fig. 1, serums C3 and C4 showed a significant positively correlation with each other (*r* = 0.726, *P* < 0.001). The level of serum C3 was negatively correlated with number of involved organs (*r* = − 0.441, *P* < 0.001), IgG (*r* = − 0.362, *P <* 0.001), IgG3 (*r* = − 0.338, *P* < 0.001), and IgG4 (*r* = − 0.425, *P* < 0.001), whereas positively correlated with IgA (*r* = 0.341, *P* < 0.001). Similarly, serum C4 level was negatively correlated with number of involved organs (*r* = − 0.309, *P* < 0.001), laboratory parameters such as IgG (*r* = − 0.436, *P* < 0.001), IgG1 (*r* = − 0.315, *P* < 0.001), IgG3 (*r* = − 0.301, *P* < 0.001), and IgG4 (*r* = − 0.422, *P* < 0.001). In addition, serum C3 was weakly correlated with the age (*r* = − 0.162, *P* = 0.005), IgG4-RD RI (*r* = − 0.201, *P* = 0.005), IgG1 (*r* = − 0.216, *P* < 0.001), and hsCRP (*r* = 0.203, *P* = 0.002). Serum C4 was weakly correlated with IgG4-RD RI (*r* = − 0.207, *P* < 0.001), hsCRP (*r* = 0.192, *P* = 0.003), and IgA (*r* = 0.224, *P* < 0.001).

### Treatment efficacy in IgG4-RD with hypocomplementemia

All patients with hypocomplementemia were treated with glucocorticoids (GCs), GCs combined with immunosuppressant agents (GCs plus IM) or GCs combined with rituximab (RTX). The standard induction dosage of oral prednisone was 0.5–1.0 mg/kg/day in the first month and tapered per 1 or 2 weeks to the maintenance dosage. 18 (27.7%) patients received GC monotherapy. One (1.5%) patient received GCs plus RTX. Others were treated with GCs plus IM, including cyclophosphamide (CYC) (*n* = 22, 33.8%), mycophenolate mofetil (MMF) (*n* = 12, 18.5%), methotrexate (MTX) (*n* = 5, 7.7%), iguratimod 5 (*n* = 5, 7.7%), and leflunomide (*n* = 1, 1.5%).

**Fig. 2:**
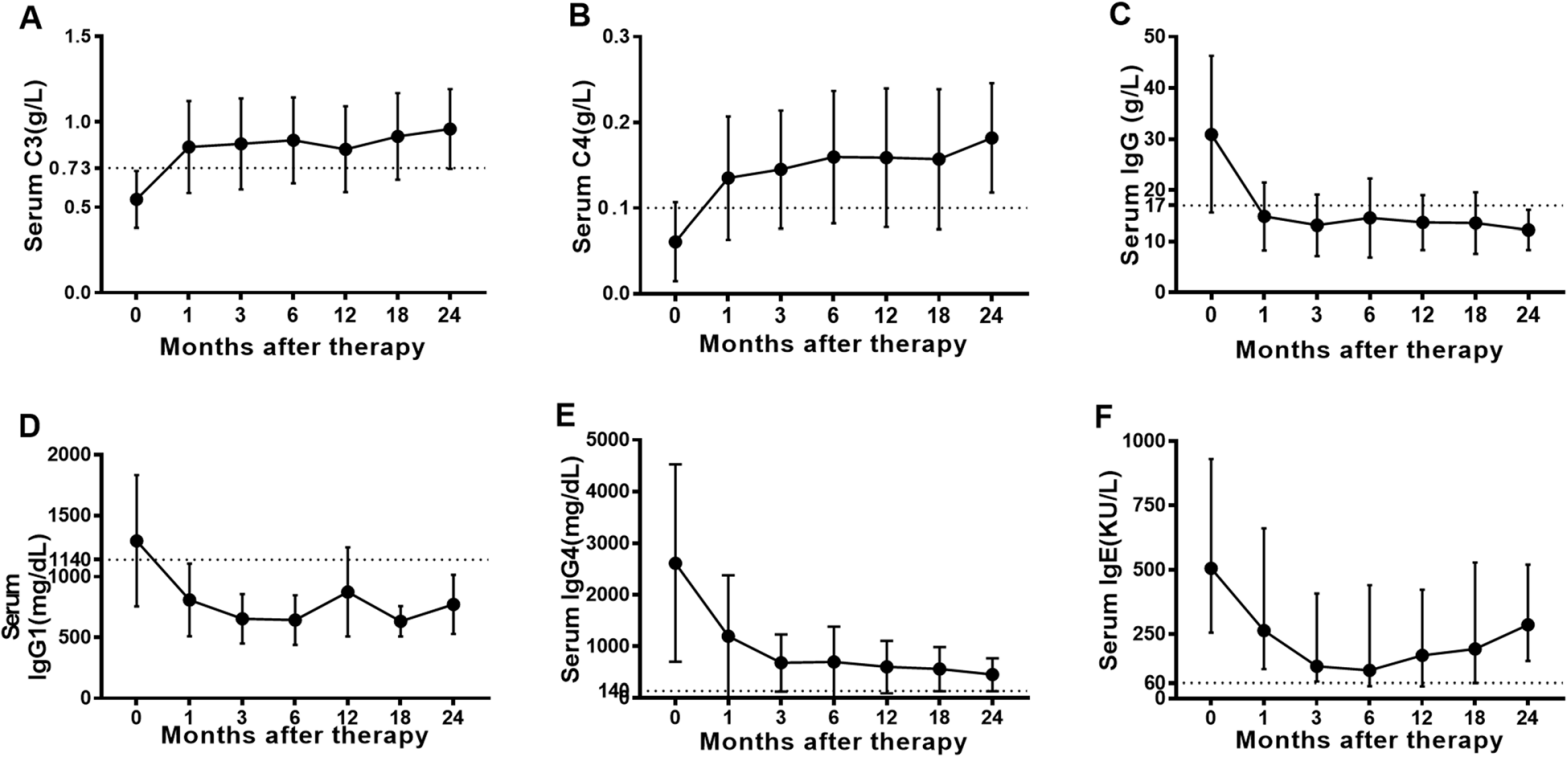
Changes of serums C3 and C4, other laboratory parameters and IgG4-RI after treatment in IgG4-RD patients with hypocomplementemia. Change of serum C3 (**a**), serum C4 (**b**), serum IgG (**c**), serum IgG1 (**d**), serum IgG4 (**e**), and serum IgE (**f**) after treatment in IgG4-RD patients with hypocomplementemia

The average follow-up time of IgG4-RD patients with hypocomplementemia was 34.01 ± 18.34 months. The level of serums C3 and C4 increased to the normal range the first month after treatment (Fig. 2a, b). Laboratory parameters such as ESR, hsCRP, IgG (Fig. 2c), IgG1 (Fig. 2d), IgG4 (Fig. 2e), and IgE (Fig. 2f) decreased significantly after treatment. Disease response occurred in 64 (98.5%) patients at month 3 and was observed quickly. One patient had no improvement at the 3rd month until increased the dosage of GCs and combined with IMs. Fifteen (23.1%) patients relapsed during follow-up with mean recurrence time 14.2 ± 13.8 months, while only 25% of them had hypocomplementemia while relapsed.

### Comparison of treatment and outcome in IgG4-RD hypocomplementemia group and normal complement group

**Fig. 3:**
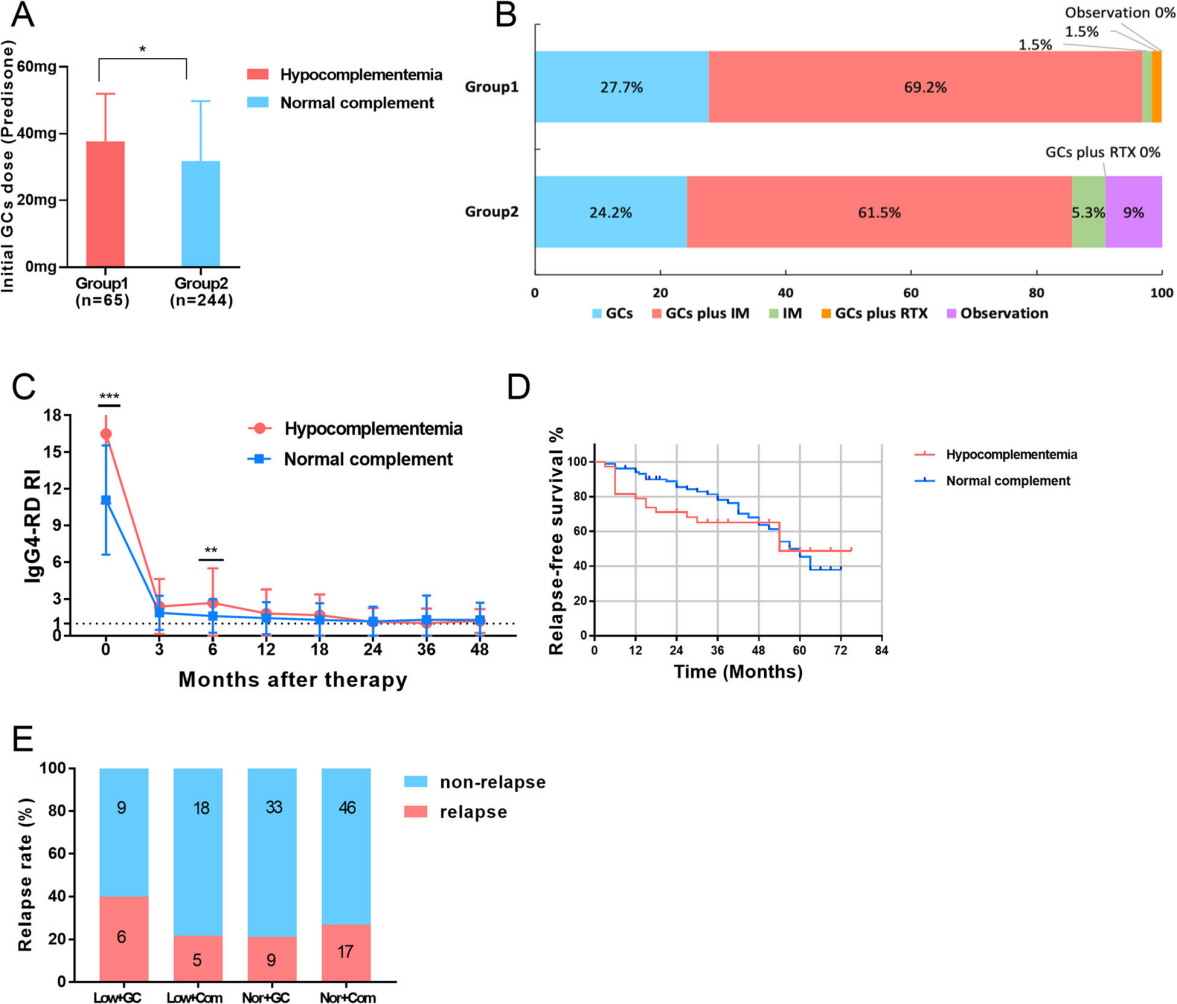
Comparison of treatment regimens ad therapeutic outcomes of IgG4-RD patients in hypocomplementemia group and normal complement group. **a** Comparison of initial GCs dose between hypocomplementemia group (group 1) and normal complement group (group 2). **b** The first-line therapies for hypocomplementemia group (group 1) and normal complement group (group 2). **c** The changes in IgG4-RD RI scores during the 48 months of follow-up in hypocomplementemia group (*n* = 38) and normal group (*n* = 105). **P < 0.01, ***P < 0.001. **d** Kaplan-Meier survival analysis suggested no significant difference of relapse-free survival between two groups(*P* = 0.401). **e** Relapse rates of four subgroups were compared. Low+GC hypocomplementemia group treated with GCs, Nor+GC normal complement group treated with GCs, Low+Com hypocomplementemia group treated with combined therapy (GCs plus IM), Nor+Com normal complement group treated with treated with combined therapy (GCs plus IM). Chi-Square test showed no statistical difference in four subgroups

We compared the first-line treatment between the two groups. The initial doses of GCs in hypocomplementemia group were higher than the normal complement group (37.77 ± 14.28 vs 31.71 ± 18.1, *P* = 0.013) (Fig. 3a). GCs-based therapies (GCs alone or in combination with IM/RTX) were used more frequently in hypocomplementemia group (100% vs 85.7%, *P* = 0.001). Higher IgG4-RI and more organs involved in hypocomplementemia group, indicating that the different treatment regimens between the two groups.

To compare therapeutic outcomes of hypocomplementemia group and normal complement group, 38 patients with hypocomplementemia and 105 patients with normal complement who were initially treated with initial GC alone or in combination with IMs, with initial GC doses of 0.5–1.0 mg/kg/ day (30–60 mg/day) of prednisone equivalent, and followed-up more than 24 months were included. The results showed that although IgG4-RD RI score was higher in hypocomplementemia group at baseline and 6th month follow-up (*P* < 0.001 and *P* = 0.005, respectively), there was no significant difference between two groups at 12, 24, 36, and 48 months after therapy (Fig. 3c). Relapse rate was observed in in 21.9%, 29.0%, 29.0%, 35.0%, and 51.2% of the patients with hypocomplementemia at 12, 24, 36, 48, and 60 months after therapy, respectively. Kaplan-Meier survival analysis suggested no significant difference of relapse-free survival between two groups (*P* = 0.401) (Fig. 3d). Relapse rates were analyzed in four subgroups after adjustment to hypocomplementemia patients treated with GC monotherapy group (*n* = 15), hypocomplementemia patients treated with combined therapy group (*n* = 23), normal complement GCs treated with monotherapy group (*n* = 42), and normal complement treated with combined therapy group (*n* = 63). The relapse rates of four subgroups within 24 months were 40.0%, 21.7%, 21.4%, and 27%, respectively. The relapse rate of hypocomplementemia GC monotherapy group was higher than hypocomplementemia combination therapy group (*P* = 0.225) without statistical difference, as well as normal complement GC monotherapy group (*P* = 0.161) (Fig.3e).

## Discussion

As far as we know, this is the first study to elaborate the clinical and laboratory characteristics, treatment response, and prognosis of IgG4-RD with hypocomplementemia. Further, we investigated serum C3 and C4 would be biomarkers for disease activity.

In our prospective cohort, complements C3 and/or C4 were decreased in one fifth of the IgG4-RD patients. Compared with normal complement group, IgG4-RD with hypocomplementemia was a distinct clinical phenotype, which with higher number of involved organs, higher disease activity, and disparities of involved organs. John Stone suggested that IgG4-RD consisted of two overlapping subsets: a proliferative type and fibrotic type. Patients with the proliferative subset of IgG4-RD tend to have disease affecting the glandular and epithelial tissues and have high serum concentrations of IgG4, IgG1, and IgE; a higher likelihood of hypocomplementemia [15]. Our study demonstrated that IgG4-RD with hypocomplementemia had higher incidence of lymphadenopathy, dacryoadenitis, sialadenitis, autoimmune pancreatitis, lung disease, paranasal sinusitis, sclerosing cholangitis, and prostate gland involvement, compared with normal complement group. Parameters associated with inflammatory and high disease activity include count of eosinophil, ESR, IgG, IgE, IgG1, IgG3, and IgG4 were significantly higher. Therefore, we think that hypocomplementemia is one of the most important features of proliferative subset.

As we known, hypocomplementemia is one important inclusion criteria associated with kidney involvement in 2019 American College of Rheumatology/European League Against Rheumatism classification criteria for IgG4-RD [16].  Kawano reported more than 50% of patients with active IgG4-tubulointerstitial nephritis (TIN) which is the most common manifestation of IgG4-RD with kidney involvement had hypocomplementemia [17]. However, in our cohort, there was no significant difference in renal involvement between hypocomplementemia and normal complement groups. Since apart from IgG4-TIN, IgG4-related glomerular nephritis, renal parenchymal nodule lesions, and renal pelvis involvement were considered as renal involvement in our study. As Teng et al. studied 65 IgG4-related urinary disease (RUD) patients, TIN only accounted for 21 (32.3%) of IgG4-RUD and the mean serum C3 level of TIN group was significantly lower than other groups. The mean serums C3 and C4 were normal in a group of renal pelvis or ureter involvement, abnormal renal radiological findings, and renal parenchymal lesions accompanied by retroperitoneal fibrosis [18].

We compared treatment outcomes between two groups. After 1-month treatment, the average level of serums C3 and C4 in hypocomplementemia group recovered swiftly. Hypocomplementemia at baseline would not be the predictor for prognosis as there was no significant difference of relapse rate within 72 months in two groups after treatment. However, considering that patients with hypocomplementemia were given more aggressive treatment because of the higher disease activity, the predictive value of hypocomplementemia for prognosis of IgG4-RD in this study may be biased. We compared relapse rates in four subgroups after matching treatment. The relapse rate of hypocomplementemia with GC monotherapy group was 40.0% higher than other groups, while there was no statistics difference possible due to the small sample size. Prospective cohort study with larger sample size is needed.

Mechanism of complement activation in IgG4-RD remains not clear. It was reported that anti-galectin-3 [19], anti-annexin A11 [20], anti-laminin-511 [21], and anti-prohibitin [22] had been detected in a minority of patients with IgG4-RD, for example, anti-galectin-3 antibodies were identified in approximately 30% of a cohort of 121 IgG4-RD patients with multiple organ involvement [19]. Therefore, antigen-antibody immune complex may play a role in activating complement pathway. Muraki et al. proposed that based on the high serum circulating immune complex in autoimmune pancreatitis, the classical complement activation pathway is thought to be involved in IgG4-RD [23].  As we all known, IgG4 molecule does not bind complement effectively and is unable to activate complement pathway, and one plausible explanation is elevated IgG1 played a prominent role via the classical complement pathway [1, 24]. As we also found IgG1 elevated remarkably in hypocomplementemia group and serums C3 and C4 were negatively correlated with IgG1. After treatment, IgG1 decreased along with the increase of serums C3 and C4 to normal in the first month, whereas the decline of IgG4 lagged behind. Another potential explanation is that IgG4 may activate the complement system through the MBL pathway [24, 25]. Sugimoto et al. reported that high serum levels of C1q-binding IgG4 in IgG4RD patients with hypocomplementemia. They observed marked reduction of total complement hemolytic (CH50) and complement activity in the classical complement pathway as well as the MBL pathway in normal human serum incubated with polyethylene glycol precipitates-immune complexes isolated from IgG4RD patients with hypocomplementemia [25]. Altered glycosylation of IgG1 and IgG4 antibody subclasses might also have a role in causing hypocomplementemia in patients with IgG4-RD [26].  Interestingly, IgA in hypocomplementemia group was significantly lower than normal complement group and positively correlated with serum C3 and C4 level. The role of IgA in the complement activation pathway remains a mystery.

## Conclusion

In conclusion, IgG4-RD patients with hypocomplementemia compared to normal complement patients had higher disease activity, higher number of affected organs and proliferative subtype features. The levels of serum C3 and C4 were negatively correlated with indicators of disease activity number of involved organs and laboratory parameters such as IgG, IgG3, and IgG4, and implicated serums C3 and C4 could be the biomarkers for disease activity. The low level of serums C3 and C4 could be recovered quickly after immunosuppressive therapy. IgG4-RD patients with hypocomplementemia respond well to treatment and have no significant difference of relapse rate in IgG4-RD patients with normal complement.

## Data Availability

The datasets used and/or analyzed during the current study are available from the corresponding author on reasonable request.

## Abbreviations

IgG4-RD: Immunoglobulin G4-related disease
RI: Responder index
IgG: Immunoglobulin G
hsCRP: Hypersensitive C-reactive protein
MBL: Mannan-binding lectin
SLE: Systemic lupus erythematosus
APS: Antiphospholipid syndrome
AAV: Anti-neutrophil cytoplasmic antibody associated vasculitis
CT: Computed tomography
MRI: Magnetic resonance imaging
PET/CT: Positron emission tomography/computed tomography
GCs: Glucocorticoids
IM: Immunosuppressant
RTX: Rituximab
CYC: Cyclophosphamide
MMF: Mycophenolate mofetil
MTX: Methotrexate
IQR: Interquartile range
HR: Hazard ratio
TIN: Tubulointerstitial nephritis
RUD: Urinary disease
